# A systematic review and meta-analysis of prevalence of vitamin D deficiency among Indonesian pregnant women: a public health emergency

**DOI:** 10.1016/j.xagr.2023.100189

**Published:** 2023-03-12

**Authors:** Gilbert Sterling Octavius, Vamela Adman Daleni, Glenda Angeline, Cindy Virliani

**Affiliations:** 1 Department of Pediatrics, Universitas Pelita Harapan, Tangerang, Indonesia (Dr Octavius); 2 Department of Obstetrics and Gynecology, Universitas Pelita Harapan, Tangerang, Indonesia (Drs Adman Daleni, Angeline, and Virliani)

**Keywords:** hypovitaminosis D, Indonesia, prevalence, public health emergency, vitamin D deficiency

## Abstract

**OBJECTIVE:**

There are few in-depth investigations or meta-analyses determining the prevalence of vitamin D insufficiency in expectant Indonesian mothers. This systematic review and meta-analysis aims to determine this prevalence.

**DATA SOURCES:**

We searched information using the following databases: MEDLINE, PubMed, Google Scholar, Cochrane Library, ScienceDirect, Neliti, Indonesia Onesearch, Indonesian Scientific Journal Database, bioRxiv, and medRxiv.

**STUDY ELIGIBILITY CRITERIA:**

The inclusion criteria included cross-sectional studies or observational studies published in any language, studying Indonesian pregnant women whose vitamin D levels were measured.

**METHODS:**

Vitamin D deficiency in this review was defined as serum 25-hydroxyvitamin D <50 nmol/L, whereas vitamin D insufficiency was defined as serum 25-hydroxyvitamin D between 50 and 75 nmol/L. The analysis was done using Stata software with the Metaprop command.

**RESULTS:**

The meta-analysis included 6 studies involving 830 pregnant women aged 27.6–30.6 years. Prevalence of vitamin D deficiency among Indonesian pregnant women was 63% (95% confidence interval, 40–86; *I*^2^, 98.9%; *P*<.0001). The prevalence rates of vitamin D insufficiency and hypovitaminosis D were 25% (95% confidence interval, 16–34; *I*^2^, 83.37%; *P*<.01) and 78% (95% confidence interval, 60–96; *I*^2^, 96.81%; *P*<.01), respectively. The mean serum vitamin D level was 40.59 nmol/L (95% confidence interval, 26.04–55.13; *I*^2^, 99.57%; *P*<.01).

**CONCLUSION:**

Pregnant women in Indonesia are at risk for vitamin D deficiency, which constitutes a public health issue. Possible unwanted consequences, including preeclampsia and small-for-gestational-age newborns, are more likely to occur when vitamin D deficiency in pregnant women is left untreated. However, more studies are needed to prove these relationships.

**AJOG MFM at a Glance**

**Why was this study conducted?**

The prevalence of vitamin D deficiency (VDD) in Indonesian pregnant women is mostly unknown. No extensive epidemiologic studies or meta-analyses have been conducted to assess the prevalence of VDD in Indonesian pregnant women.

**Key findings**

The prevalence of VDD in Indonesian pregnant women is 65%. The prevalence rates of vitamin D insufficiency and hypovitaminosis D are 25% and 78%, respectively. The mean serum vitamin D level is 40.59 nmol/L, lower than the <50 nmol/L cutoffs for diagnosing VDD.

**What does this add to what is known?**

Pregnant women are vulnerable to VDD, and our data confirm the finding in Indonesia. This result disproves the myth that VDD is sparse in Indonesia because of the tropical climate.

## Introduction

Vitamin D consists of 2 active metabolites, calcidiol 25-hydroxyvitamin D (25[OH]D) and calcitriol (1,25[OH]_2_D), which have proven skeletal benefits, such as preventing the development of rickets and osteomalacia.^1^^,^^2^ Illnesses such as asthma and upper respiratory tract infections, cancer, autoimmune disorders, falls, and chronic diseases of adulthood, including osteoporosis and cardiovascular disease, are all linked to low vitamin D level.^2^^,^^3^ The numerous vitamin D benefits may be attributable to the widespread presence of vitamin D receptors, except in mature muscle, including the heart and liver.^4^

Vitamin D deficiency during pregnancy has been linked to increased risk of some conditions affecting both the mother and the fetus or newborn, including preterm birth (if vitamin D deficiency occurs in the middle of pregnancy),^5^ spontaneous pregnancy loss,^6^^,^^7^ small-for-gestational-age,^8^ low birthweight,^9^ and preeclampsia.^10^^,^^11^ However, despite the importance of vitamin D during pregnancy, Indonesia does not have adequate data on vitamin D levels among pregnant women.

One study mentions that vitamin D deficiency in Indonesian pregnant women ranges from 60% to 95%,^12^ whereas another study quotes 20% to 95%.^1^ This number, however, is derived from other primary studies without any pooling. Therefore, this prevalence of vitamin D deficiency in Indonesian pregnant women is estimated at best. To our best knowledge, no large studies (such as the South East Asian Nutrition Survey [SEANUTS] in children^13^) or meta-analyses have yet been conducted on the prevalence of vitamin D levels in Indonesia. Given that Indonesia's maternal mortality ratio (MMR) is still high, at 177 per 100,000 live births, this absence of data is particularly worrisome.^14^ Although no causal links can be derived between vitamin D deficiency and MMR in Indonesia, it is essential to take action regarding vitamin D deficiency in Indonesia after realizing the adverse effects on mothers and newborns. The data on vitamin D deficiency among Indonesian pregnant women must be known so that the government, stakeholders, and clinical health practitioners can make recommendations.

Therefore, this systematic review and meta-analysis aims to determine the prevalence of vitamin D deficiency among pregnant women in Indonesia. Our secondary goal is calculating the average vitamin D levels, the prevalence of low vitamin D levels, and the prevalence of hypovitaminosis D among Indonesian pregnant women.

## Methods

### Eligibility criteria

The PRISMA (Preferred Reporting Items for Systematic Reviews and Meta-Analyses) 2020 guidance was followed.^15^ The study protocol was recorded in PROSPERO (International Prospective Register of Systematic Reviews) under registration number CRD42022347207.

The studied population was all Indonesian pregnant women with known serum vitamin D levels irrespective of gestational age during serum sampling because of our prediction of small amounts of studies in this category. The primary outcome of this study was the prevalence of vitamin D deficiency among pregnant women in Indonesia. Different studies using the same data set were considered as 1 study, and we chose 1 representative study on the basis of the required data, the completeness of the data, or both. The study's secondary outcome was mean blood vitamin D levels in Indonesian pregnant women. In this meta-analysis, there were no interventional or control groups.

Inclusion requirements included cross-sectional research or observational studies published in any language. Vitamin D deficiency in this review was defined as serum 25[OH]D <50 nmol/L, whereas vitamin D insufficiency was defined as serum 25(OH)D between 50 and 75 nmol/L.^16^ We defined hypovitaminosis D as a combination of vitamin D deficiency and vitamin D insufficiency. However, using this cutoff as a strict criterion for inclusion would result in very few studies being included in our meta-analysis. Hence, we decided to include all studies, irrespective of the cutoffs used in each study. A subgroup analysis of serum 25(OH)D cutoffs (<50 nmol/L vs not <50 nmol/L) was undertaken to assess their impact on the prevalence. In addition, we performed subgroup analyses based on: the machines used to quantify serum vitamin D and vitamin D sufficiency and deficiency; the quality of the studies; and the likelihood of bias. All supplemental resources were searched to ensure that all data were fully extracted. Case reports, case series, cohort studies, reviews, animal studies, and studies conducted outside of Indonesia were excluded from this investigation. We also excluded studies with <50 samples to maintain the stability of the prevalence pool.^17^ Another exclusion criterion were studies that did not provide enough data to calculate the prevalence rate. Finally, expectant mothers with comorbid conditions such as preeclampsia were excluded. Citations from review studies were searched to ensure literature saturation. To ensure that all relevant research was included, we also conducted manual searches and citation checks on omitted papers. We multiplied the mean values in nanogram per milliliter (ng/mL) by 2.5 for conversion to nanomole per liter (nmol/L) to retain data consistency.

### Search strategy and study selection

The literature search began and was completed on July 10, 2022. We searched 5 academic databases: MEDLINE, PubMed, Google Scholar, Cochrane Library, and ScienceDirect. Three Indonesia-specific databases—Neliti, Indonesia Onesearch, and the Indonesian Scientific Journal Database (ISJD)—were also used to increase literature saturation. We also searched bioRxiv and medRxiv for possible preprints related to our meta-analysis. The search was conducted in English and Bahasa Indonesia. The key words used were related to vitamin D (“Vitamin D deficiency,” “Vitamin D insufficiency,” “hypovitaminosis D,” “ergocalciferol,” “25-hydroxyvitamin D”), pregnancy, and Indonesia. The Medical Subject Headings (MeSH) terms for each database are listed in Supplemental Table 1. All records were entered into the Rayyan program (Rayyan Systems Inc., Cambridge, MA), which inspected them and automatically identified duplicates.^18^ This program can be used to collaboratively select relevant research. Initial searches were carried out independently by 2 authors (G.S.O. and C.V.), who then imported all of the results into Rayyan software. The first search was then cross-checked by 2 other authors (G.A. and V.A.D.). These 4 authors individually reviewed each available article. Disputes were settled through discussions. Grey literature was also actively sought, including posters, abstracts, and white papers.

### Data extraction and quality assessment

Two authors (G.S.O. and V.A.D.) independently extracted the data, which were then checked for accuracy by a third author (C.V.). We gathered pertinent data, including study identity (author and publication year), study characteristics (place, study design, and study duration), and the prevalence of vitamin D deficiency (total number of pregnant women checked for vitamin D as well and the number of pregnant women who suffered from vitamin D deficiency).

The Newcastle–Ottawa scale (NOS) for cross-sectional studies was used to rate the quality of the studies. On the NOS, a score of 7 to 9 indicates good study quality, a score of 4 to 6 moderate or fair quality, and a score of 0 to 3 poor quality.^19^ We used JBI criteria for prevalence studies to evaluate risk of bias. Studies with a score of 0 to 3 were considered as low-risk, a score of 4 to 6 as moderate-risk, and a score of 7 to 9 as high-risk.^20^ The NOS and JBI scale were separately evaluated by 3 reviewers (G.S.O., G.A., and V.A.D.), and C.V. double-checked the results. All differences were resolved through discussion until an agreement was reached. Emails or ResearchGate messages were sent to corresponding authors to inquire about any missing or additional data.

### Data synthesis

We calculated the point prevalence by dividing the total number of women with known vitamin D levels by the number of pregnant women who had vitamin D deficiency.^21^ The analysis was done using Stata software, Version 17.0 (StataCorp, College Station, TX), and Metaprop was the command of choice for prevalence calculation. The DerSimonian and Laird random-effects model was chosen, and we calculated the 95% confidence interval (CI) using the Clopper–Pearson method.^22^ We used prediction intervals to assess heterogeneity if >10 studies were included,^23^ and between-study heterogeneity was explored with a Galbraith plot.^24^ Small-study effects were assessed with funnel plot analysis if >10 studies were included,^25^ and Begg and Mazumdar test was used for rank correlation,^26^ and the Egger test for a regression intercept.^27^ If the funnel plot was asymmetrical, trim-and-fill analysis was conducted.^28^ Random effects with Hartung–Knapp–Sidik–Jonkman method were used to calculate the weighted mean 25(OH)D values.^29^ We also conducted a sensitivity analysis to assess one study's impact on the overall prevalence.^30^

## Results

**Figure 1:**
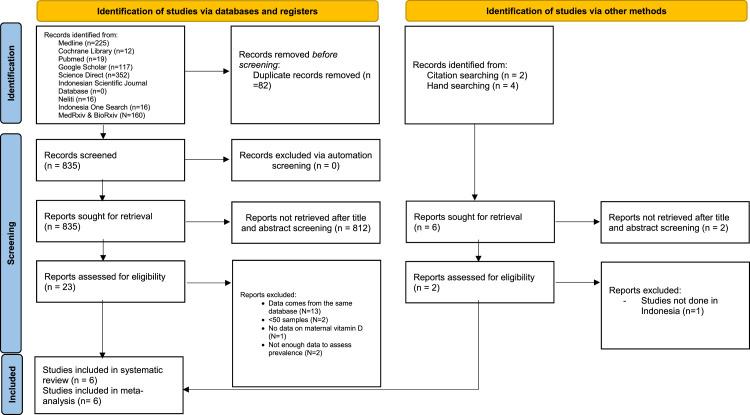
PRISMA flowchart for selection of included studies *PRISMA*, Preferred Reporting Items for Systematic Reviews and Meta-Analysis.

Of the 917 identified articles, 82 were duplicate content. After title and abstract evaluation, 23 publications underwent a thorough evaluation and were excluded, and 812 records were left. Five articles remained after applying exclusion criteria. Another 6 publications were identified through citations and manual searches, of which 1 was included in the final analysis. Thus, 6 articles were included in this systematic review and meta-analysis (Figure 1). The Table lists the study characteristics and the scores on the NOS, whereas Supplemental Table 2 lists the risk of bias for each study. Supplemental Table 3 summarizes all exclusions of important studies and their justifications.

Octavius. A systematic review and meta-analysis of prevalence of vitamin D deficiency. Am J Obstet Gynecol Glob Rep 2023.

**Table:** Characteristics of included studies

Author, y	Study period	Study design	Mean age (y)	Gestational age criteria for inclusion	Location	Mean vitamin D level (nmol/L)	Vitamin D estimation method	Vitamin D cutoff	Total participants with measured vitamin D	Newcastle Ottawa Scale				
										Selection	Comparability	Outcome	Total	Classification
Ilmiawati et al,^31^ 2020	Feb–June 2019	Cross-sectional	30.6 (5.03)	Third trimester	Minangkabau	57.5 (25)	ELISA	Deficient (<50 nmol/L), insufficient (50–79 nmol/L), and sufficient (≥80 nmol/L)	88	2	1	3	6	Moderate
Judistiani et al,^32^ 2019	July 2016–2019	Cross-sectional	28.4 (5.83)	10–14 wk	Bandung, Sukabumi, Cimahi, and Waled	36.75 (16.25)	ELISA	Deficient (< 50 nmol/L), insufficient (50–74.9 nmol/L), and normal (≥75 nmol/L)	204	3	2	3	8	Good
Irwinda and Andardi,^33^ 2020	Jan 2017–Aug 2019	Cross-sectional	29.1 (6.6)	After delivery	Jakarta	42.89 (23.88)	LCMS	Deficient (< 50 nmol/L) and normal (≥50 nmol/L)	60	2	1	2	5	Moderate
Aji et al,^34^ 2020	June 2017–April 2018	Cross-sectional	29.77 (5.69)	At least 13 wk	Padang, Padang Pariaman, Payakumbuh, Lima Puluh Kota, and Pariaman	14 (6.98)	ELISA	Deficient (< 30 nmol/L), insufficient (30–49.9 nmol/L), and normal (≥50 nmol/L)	184	2	2	3	7	Good
Wibowo et al,^35^ 2017	Feb 2012–April 2015	Cross-sectional	N/A	≤14 wk	Jakarta	29.93 (13)	CLIA	Deficient (< 75 nmol/L), insufficient (75–499 nmol/L), and normal (≥500 nmol/L)	234	2	1	3	5	Moderate
Putri et al,^36^ 2019	N/A	Cross-sectional	27.67 (4.06)	>28 wk	Kabupaten Tanah Datar	63.6 (26.2)	ELISA	N/A	60	2	0	3	5	Moderate

*CLIA*, chemiluminescent immunoassay; *ELISA*, enzyme-linked immunosorbent assay; *LCMS*, liquid chromatography–tandem mass spectroscopy; *N/A*, not available.

The oldest study was conducted by Wibowo et al^35^ between February 2012 and April 2015. The rest of the studies were done between 2016 and 2019. All of the studies used the cross-sectional method. Two studies included the measurement of serum 25(OH)D during the first trimester,^32^^,^^35^ 1 study measured serum 25(OH)D during the third trimester,^31^ and 1 study measured serum 25(OH)D after delivery.^33^ Another 2 studies measured serum 25(OH)D at <13 and >27 weeks^34^ and at 28 weeks^36^ of gestation, respectively. Three studies were conducted in West Sumatra,^31^^,^^34^^,^^36^ whereas the other 3 were done in Java island (2 studies in Jakarta^34^^,^^35^ and 1 in the Bandung region^32^).

**Figure 2:**
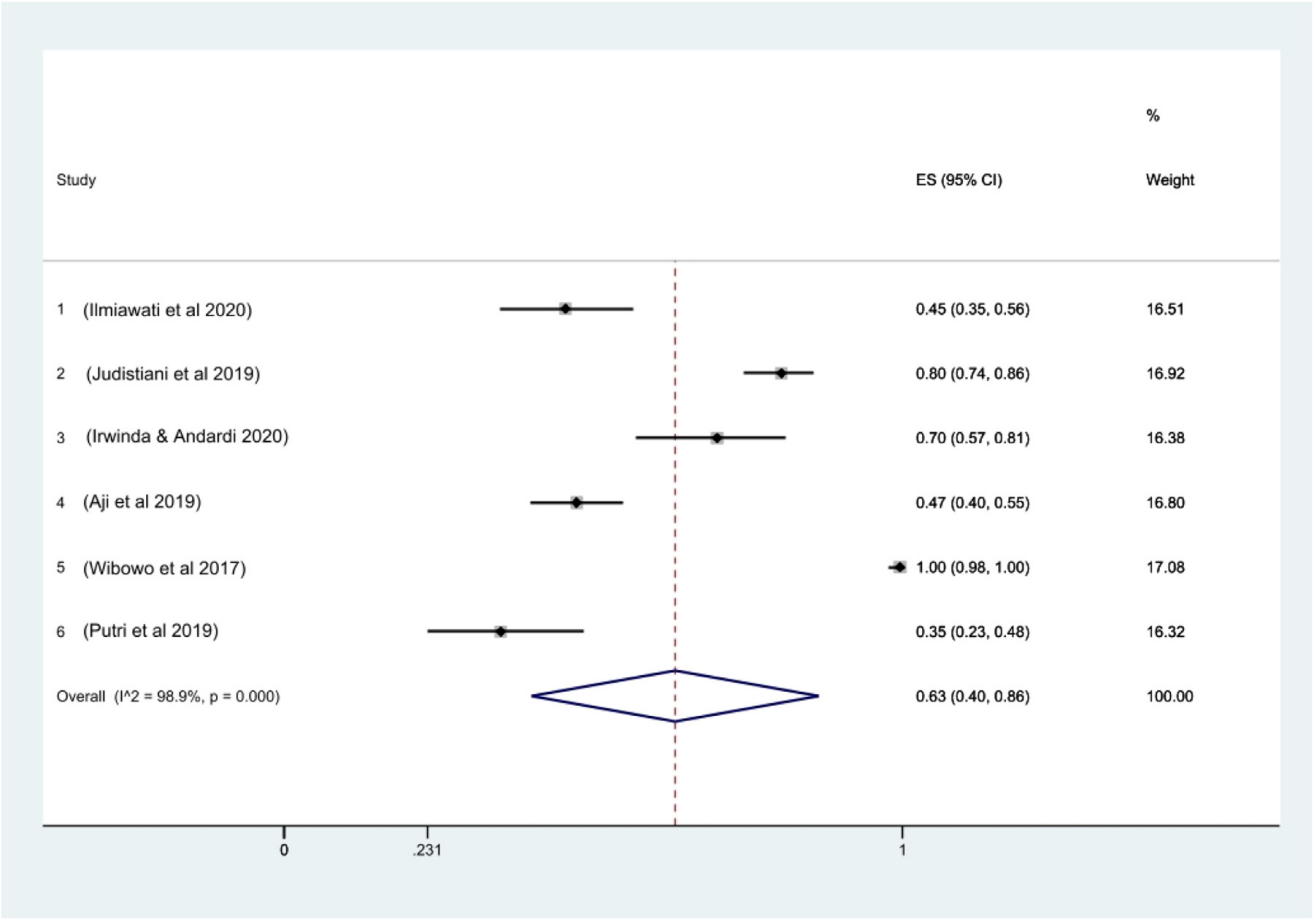
Prevalence of vitamin D deficiency in Indonesian pregnant women *CI*, confidence interval; *ES*, effect size.

A total of 830 pregnant women were included in this meta-analysis, aged from 27.6 to 30.6 years. The prevalence of vitamin D deficiency among these women was 63% (95% CI, 40–86; *I^2^*, 98.9%; *P*<.0001) (Figure 2). No heterogeneity was detected according to the Galbraith plot (Supplemental Figure 1). With a *P* value of .45, the Begg and Mazumdar rank correlation test found no indication of publication bias. With a *P* value of .0075, the Egger test for a regression intercept revealed potential publication bias. Sensitivity analyses showed that excluding studies conducted by Wibowo et al^35^ yielded a prevalence rate of 56% (95% CI, 38–74).

Octavius. A systematic review and meta-analysis of prevalence of vitamin D deficiency. Am J Obstet Gynecol Glob Rep 2023.

Subgroup analysis was performed for study quality, serum 25(OH)D cutoffs, machine used to measure vitamin D, and risk of bias. The prevalence rate was higher among studies using <50 nmol/L as their cutoff compared with those that did not (66% vs 61%). However, the CI was wider for studies that did not use <50 nmol/L as their cutoff than for those that did (16–106 vs 44–87). The heterogeneity between the groups was not significant (*P*=.853) (Supplemental Figure 2). Studies that used enzyme-linked immunosorbent assay (ELISA) had a much lower vitamin D deficiency prevalence (52%; 95% CI, 30–75) compared with studies that used non-ELISA tests (99%; 95% CI, 99–100). The heterogeneity between the groups was significant (*P*<.01) (Supplemental Figure 3). Studies of moderate quality also seemed to have lower prevalence rates of vitamin D deficiency (63%; 95% CI, 27–98) compared with those of good quality (68%; 95% CI, 64–73). However, the CI was narrower in studies of good quality. The heterogeneity between the groups was not significant (*P*=.759) (Supplemental Figure 4). Lastly, studies with low risk of bias had a higher prevalence of vitamin D deficiency (73%; 95% CI, 68–78) compared with studies with moderate risk of bias (63%; 95% CI, 28–99). The heterogeneity between the groups was not significant (*P*=.595) (Supplemental Figure 5).

**Figure 3:**
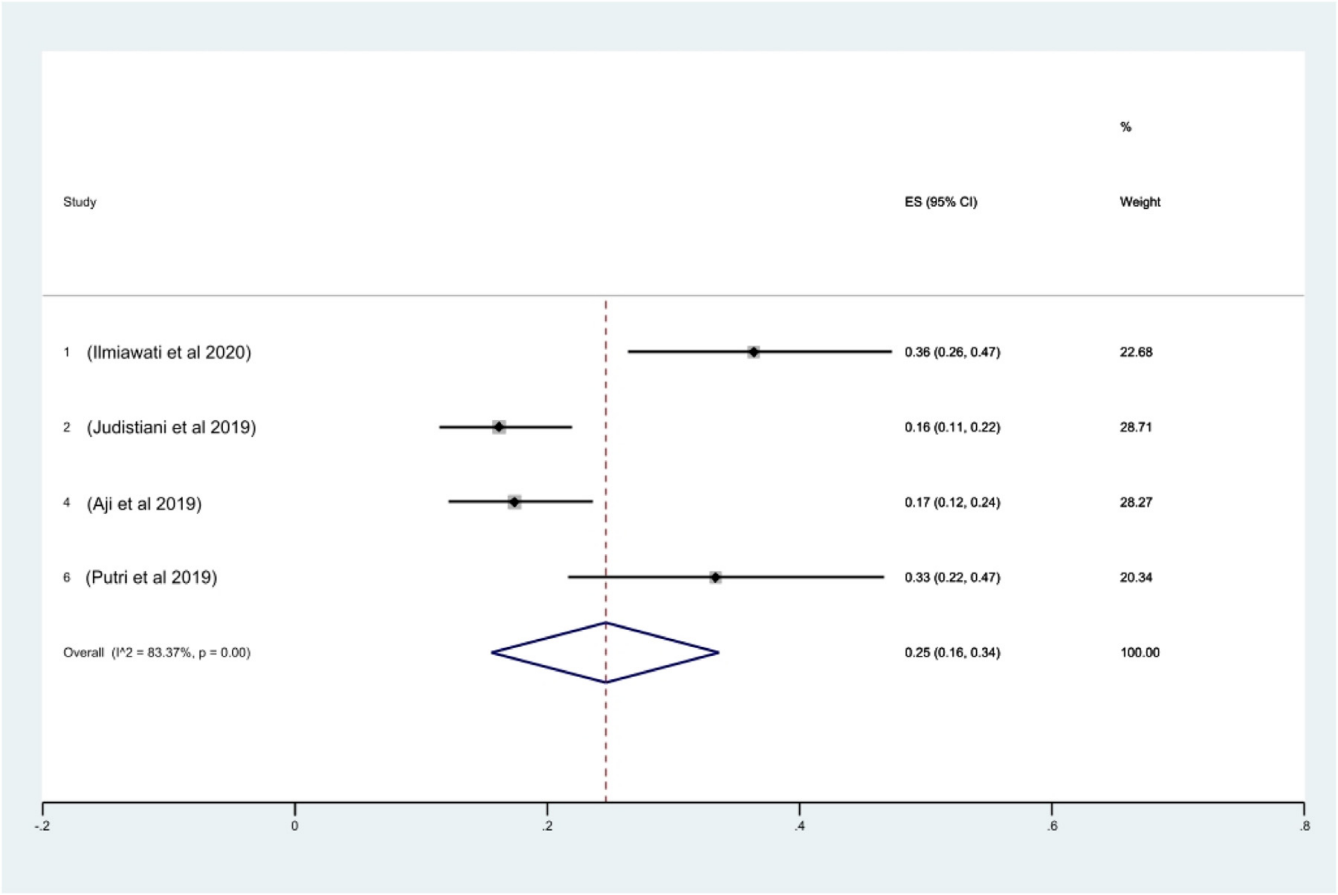
Prevalence of vitamin D insufficiency in Indonesian pregnant women *CI*, confidence interval; *ES*, effect size.

With regard to the prevalence of vitamin D insufficiency among Indonesian pregnant women, 4 studies provided sufficient data for calculation. The prevalence rate of vitamin D insufficiency was 25% (95% CI, 16–34; *I^2^*, 83.37%; *P*<.01) (Figure 3). No heterogeneity was shown by the Galbraith plot (Supplemental Figure 6). The rank correlation test by Begg and Mazumdar returned a *P* value of .31, which excludes publication bias. With a *P* value of .0001, the Egger test for a regression intercept showed some indication of publication bias. Lastly, 78% (95% CI, 60–96; *I^2^*, 96.81%; *P*<.01) of pregnant women in Indonesia suffer from hypovitaminosis D. No heterogeneity was shown by the Galbraith plot (Supplemental Figure 7). The rank correlation test by Begg and Mazumdar returned a *P* value of .73, which excludes publication bias. With a *P* value of .11, the Egger test for a regression intercept showed no sign of publication bias.

Octavius. A systematic review and meta-analysis of prevalence of vitamin D deficiency. Am J Obstet Gynecol Glob Rep 2023.

**Figure 4:**
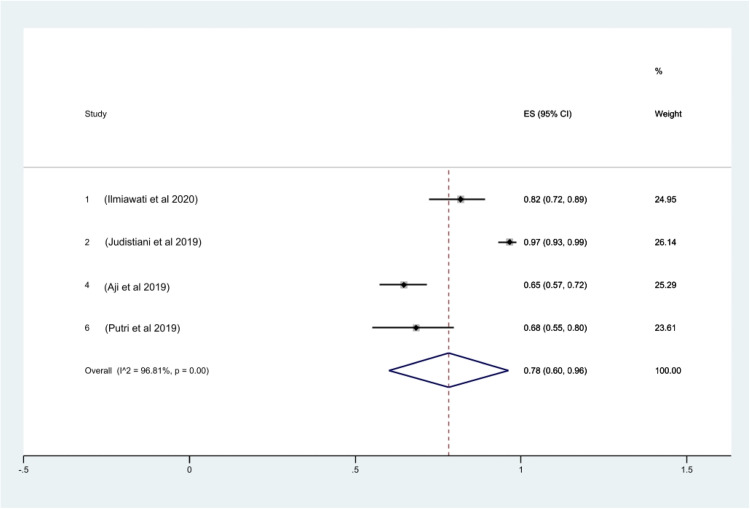
Prevalence of hypovitaminosis D in Indonesian pregnant women *CI*, confidence interval; *ES*, effect size.

Octavius. A systematic review and meta-analysis of prevalence of vitamin D deficiency. Am J Obstet Gynecol Glob Rep 2023.

**Figure 5:**
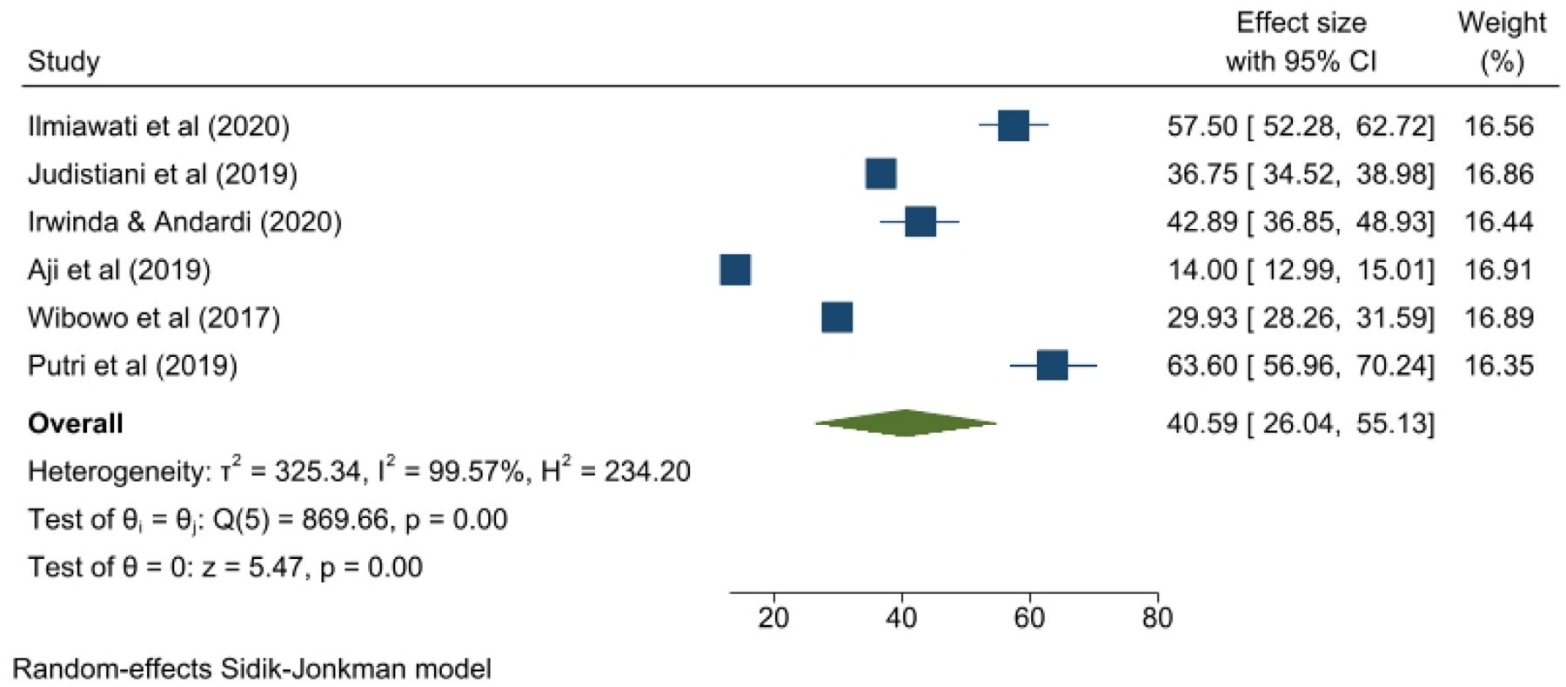
Mean vitamin D levels in Indonesian pregnant women *CI*, confidence interval.

The mean serum vitamin D level was 40.59 nmol/L (95% CI, 26.04–55.13; *I*^2^, 99.57%; *P*<.01) (Figure 5). The Galbraith plot indicated heterogeneity (Supplemental Figure 8). With a *P* value of .71, the Begg and Mazumdar rank correlation test found no indication of publication bias. With a *P* value of .0001, the Egger test for a regression intercept found evidence of potential publication bias.

Octavius. A systematic review and meta-analysis of prevalence of vitamin D deficiency. Am J Obstet Gynecol Glob Rep 2023.

## Discussion

This study found that 25%, 63%, and 78% of all Indonesian pregnant women suffer from vitamin D insufficiency, deficiency, and hypovitaminosis D, respectively. According to Roth's criteria, Indonesian pregnant women suffer from a high prevalence rate of vitamin D deficiency, constituting a public health emergency.^3^ Different studies have shown varying results compared with ours. A meta-analysis examining vitamin D deficiency in Turkey found a prevalence rate as high as 83%.^37^ However, some studies showed lower prevalence of vitamin D deficiency and insufficiency. One study in Turkey found a prevalence rate of 53.3% for vitamin D deficiency among 90 pregnant women, albeit using a different cutoff for serum 25(OH)D level.^38^ Another study including 515 Bangladeshi pregnant women at ≤20 weeks of gestation found a prevalence rate of 17.3% for vitamin D deficiency.^39^ Lastly, a large observational study in China involving 34,417 pregnant women found that only 25.1% were vitamin D–deficient, applying the same cutoff as this review.^40^

Pregnant women in Indonesia have a pooled serum vitamin D level of 40.59 nmol/L, below the threshold for serum 25(OH)D deficiency (<50 nmol/L). This value is in concordance with the high prevalence of vitamin D deficiency. For pregnant women and new mothers, a meta-analysis in Africa discovered a higher average vitamin D level of 68.46 nmol/L (95% CI, 49.91–87.01),^41^ whereas another Iranian meta-analysis found a pooled mean of 15.02 ng/mL (95% CI, 13.68–16.35) or 37.55 nmol/L (95% CI, 34.2–40.875) after conversion.^42^

There are some explanations as to why Indonesian pregnant women suffer from such a high rate of vitamin D deficiency and low overall mean serum 25(OH)D. Despite being a country that receives sunlight throughout the entire year, rapid urbanization and associated lifestyle changes may explain the high vitamin D deficiency among Indonesian pregnant women.^41^ Southeast Asian females wear traditional heavy clothing such as hijab or shari, concealing their skin from possible sources of UV light.^43^ Furthermore, Asian females are concerned with their sun exposure, and thus many intentionally attempt to avoid it, or practice preventive measures against sunburn, such as wearing sunscreen or carrying an umbrella.^44^^,^^45^ Indonesian pregnant women also do not regularly practice vitamin D supplementation because it is not made mandatory by the National Health Minister.^46^ One study found a low consumption of vitamin D and calcium among pregnant women in West Sumatra, from where 3 of the studies in this review originated.^47^ Another critical factor that may contribute to low serum 25(OH)D is the worsening air pollution in Indonesia during the past few years.^48^ High ambient air pollution levels may cause atmospheric UV-B radiation absorption, reducing the amount of UV-B radiation available to induce vitamin D synthesis in exposed skin.^44^ Ultimately, low vitamin D levels may affect the children's vitamin D levels. One meta-analysis in Indonesia found that the prevalence of vitamin D deficiency among Indonesian children is 33%.^49^

Meanwhile, some studies find that the duration of sun exposure significantly affects serum 25(OH)D levels,^50^^,^^51^ whereas others dispute this association.^52^^,^^53^ Individual response to sunlight exposure and Fitzpatrick skin types may explain these conflicting results.^54^ However, we also acknowledge the fact that different serum 25(OH)D cutoffs used in every study significantly alter the prevalence rate of vitamin D insufficiency, deficiency, and hypovitaminosis D. Given the varying characteristics among these studies and reviews, a direct comparison with other studies seems almost impossible.^55^

There are several limitations to this meta-analysis. Firstly, it was a small meta-analysis given that it involved only 6 studies,^56^ and it included 830 pregnant women, which is a small fraction of the 110-million Indonesian female population.^57^ The small number of studies precluded assessment for small-study effects and causes of heterogeneity because funnel plots and prediction intervals are not accurate if there are <10 studies.^30^^,^^58^ This may also explain the discordance among the results of the Galbraith plot, Egger test, and Begg test for heterogeneity. All tests have low power to detect heterogeneity, and coupled with the small number of studies presented in our meta-analysis, heterogeneity results are expected to be invalid.^30^^,^^59^ According to the *I^2^* index classification,^30^ our meta-analysis has substantial heterogeneity, which cannot be fully explained by study quality, serum 25(OH)D cutoffs, machines used to measure vitamin D, or risk of bias. Among these subgroups, substantial between-group heterogeneity was found only for the machine used to measure vitamin D. This supports the finding that recalibration according to type of machine yielded better results in measuring serum 25(OH)D in European populations.^60^ Heterogeneity in this study could be caused by substantial within-population variation caused by socioeconomic conditions, diet, latitude, custom, and coverage of skin with clothing, which we could not assess in this meta-analysis.^44^^,^^61^ Secondly, Wibowo et al ^35^ found a vitamin D deficiency prevalence rate of almost 100% among pregnant women, therefore skewing the overall prevalence rate. We attribute their finding to the study's moderate quality and moderate risk of bias according to NOS and JBI, respectively. Potential issues with sampling methodology, patient selection, and underpowered samples may have led to such a high prevalence rate. Hence, it is unsurprising that sensitivity analyses found an enormous pooled prevalence rate. Usually, this would decrease the confidence in the results of our pooled prevalence rate.^30^ However, even after excluding this study, the prevalence rate of vitamin D deficiency can still be considered as indicative of a public health emergency, which should be addressed urgently.^3^

Finally, there are several variables that we believe could contribute to the high prevalence of vitamin D deficiency. However, because of the paucity of data supplied in each study, we cannot evaluate them. Instead of relying on published summary measurements, a complete analysis of the factors affecting vitamin D status may have been done if individual-level data were available. Such meta-analysis of individual patient data has been hailed as the “gold standard” for meta-analysis,^62^ and it can also solve the issue of different cutoffs used by different studies. Although one meta-analysis remedied this issue using 3 different cutoffs, we could not do this because insufficient data were presented to analyze vitamin D deficiency according to 3 different cutoffs.^41^

Our work has merits despite its limitations, which justifies conducting a meta-analysis. The prevalence of vitamin D deficiency, insufficiency, and hypovitaminosis D among Indonesian pregnant women was examined. Each study's relatively recent data sets represent Indonesian pregnant women's current vitamin D status. Hence, multiple stakeholders can use this meta-analysis to plan and enforce strategies to address vitamin D deficiency in Indonesian pregnant women. Although the low number of studies may be a limitation, we have searched for all possible papers, including preprints and grey literature. Hence, this meta-analysis is a comprehensive review of vitamin D status among Indonesian pregnant women. Vitamin D research on Indonesian pregnant women may be affected by salami publication, distorting the overall results.^63^ To support this proposition, Supplemental Table 3 shows that 72% of all exclusions of studies were owing to same data sets having been used for different publications.

### Conclusion

Vitamin D deficiency is a public health emergency among Indonesian pregnant women, especially given that this population is considered a high-risk group for vitamin D deficiency. If uncorrected, vitamin D deficiency in pregnant women is passed on to the fetus and newborn, increasing the risks for unwanted complications such as small-for-gestational-age newborns and preeclampsia.

To guarantee uniformity, there has to be a more robust guiding agreement to choose the vitamin D cutoff level for each study. Deidentified, publicly accessible data must also be available to improve data accessibility and openness. There is an urgent need for a sizable population-based study that considers the phenotype and genotype of pregnant women in Indonesia, ethnic groups, geographic locations, sociocultural practices, latitude and sun exposure, dietary intake and habits, and other pertinent factors. To provide a clearer and better picture of the prevalence rate of vitamin D deficiency among pregnant women in Indonesia, conscience and research ethics should also be exercised to reduce salami publication. Institutions and funders can play a role in ensuring that publications are transparent.

Various sectors, such as the government, health practitioners, and support groups, must convene and intervene to solve this problem. Vitamin D supplementation during pregnancy might temporarily address this problem. However, until more conclusive research about timing, dosage, and supplementation combination (vitamin D alone vs vitamin D and calcium) is conducted in Indonesia, it might not be the best course of action.^64^^,^^65^

## Appendix Supplementary materials

Image, application 1

Image, application 2

## Footnotes

Supplementary material associated with this article can be found in the online version at doi:10.1016/j.xagr.2023.100189.
