# Supplementary material for: A systematic review and meta-analysis of prevalence of vitamin D deficiency among Indonesian pregnant women: a public health emergency
Source: AJOG Glob Rep. 2023 Mar 12;3(2):100189. doi: 10.1016/j.xagr.2023.100189 (PMC10205541; doi:10.1016/j.xagr.2023.100189)
Supplement: Supplementary file 1 [file mmc1.docx]

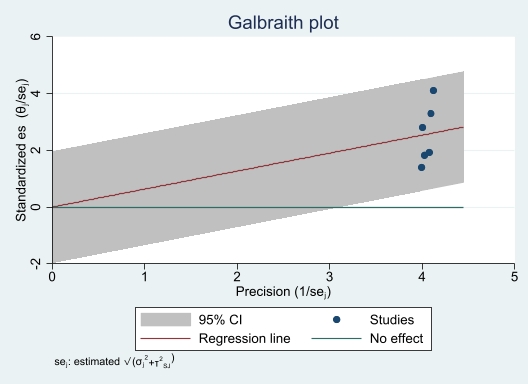


Supplementary Figure 1. Galbraith plot of vitamin D deficiency prevalence among Indonesian pregnant women


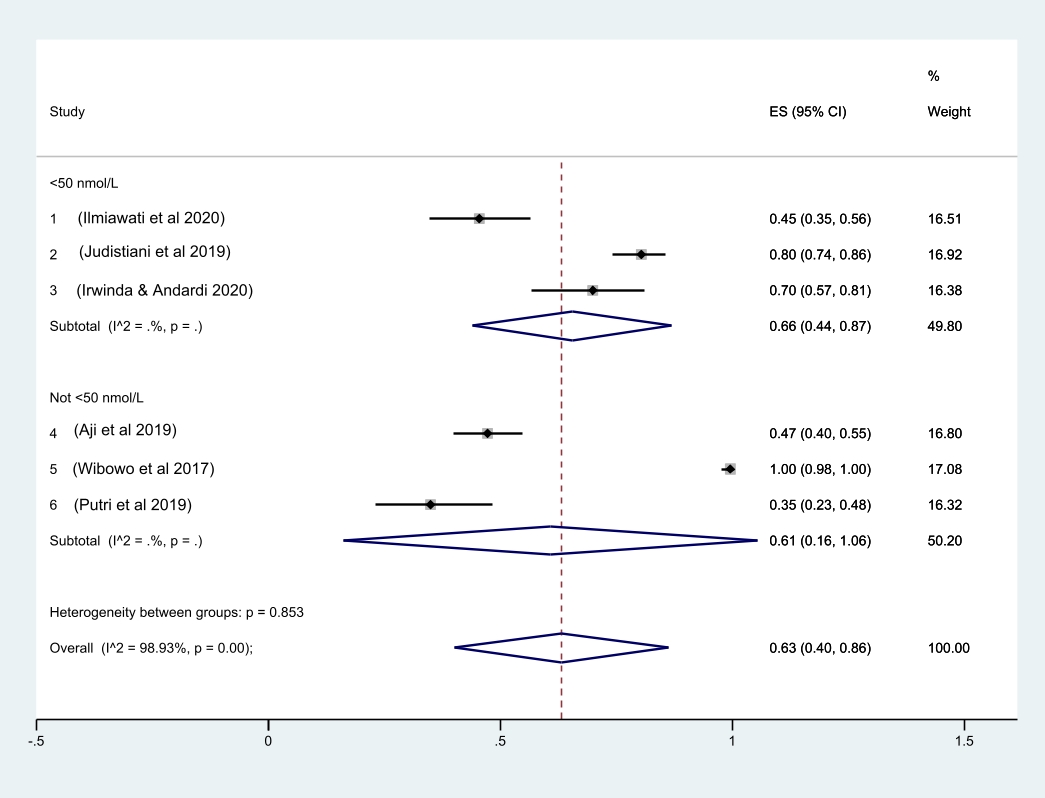


Supplementary Figure 2. Forest plot of vitamin D deficiency according to serum 25(OH)D concentration cut-off


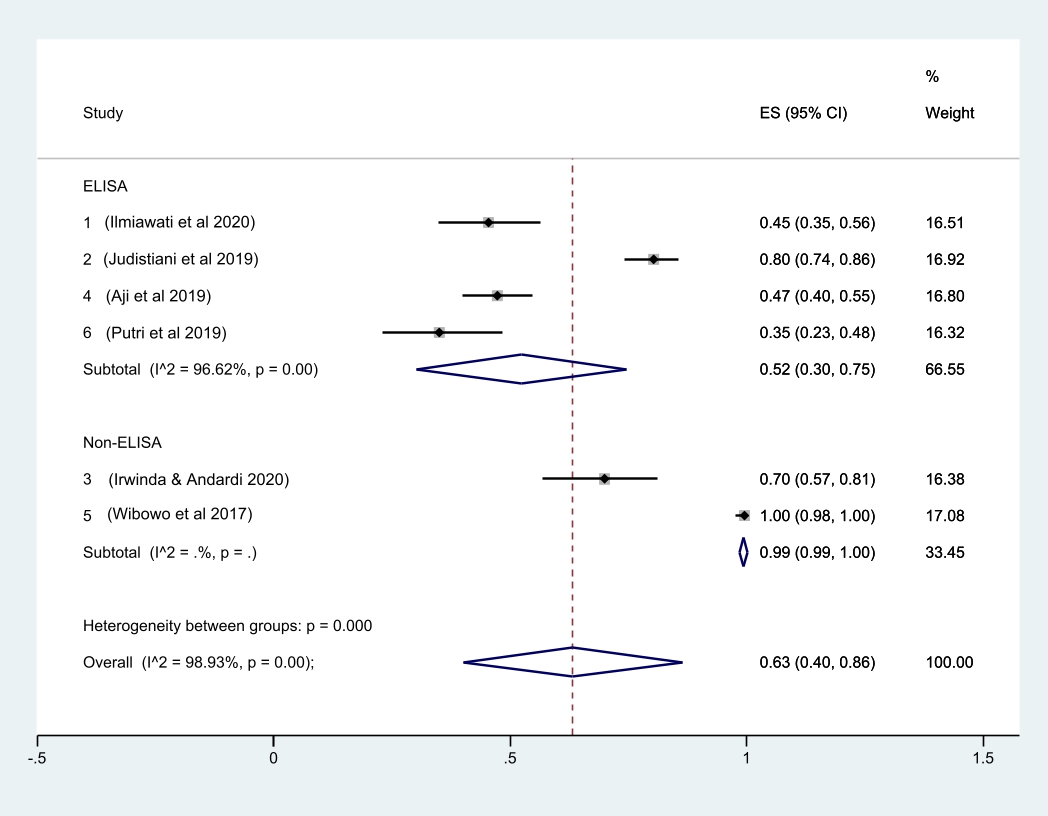


Supplementary Figure 3. Forest plot of vitamin D deficiency according to the machine used to measure serum 25(OH)D


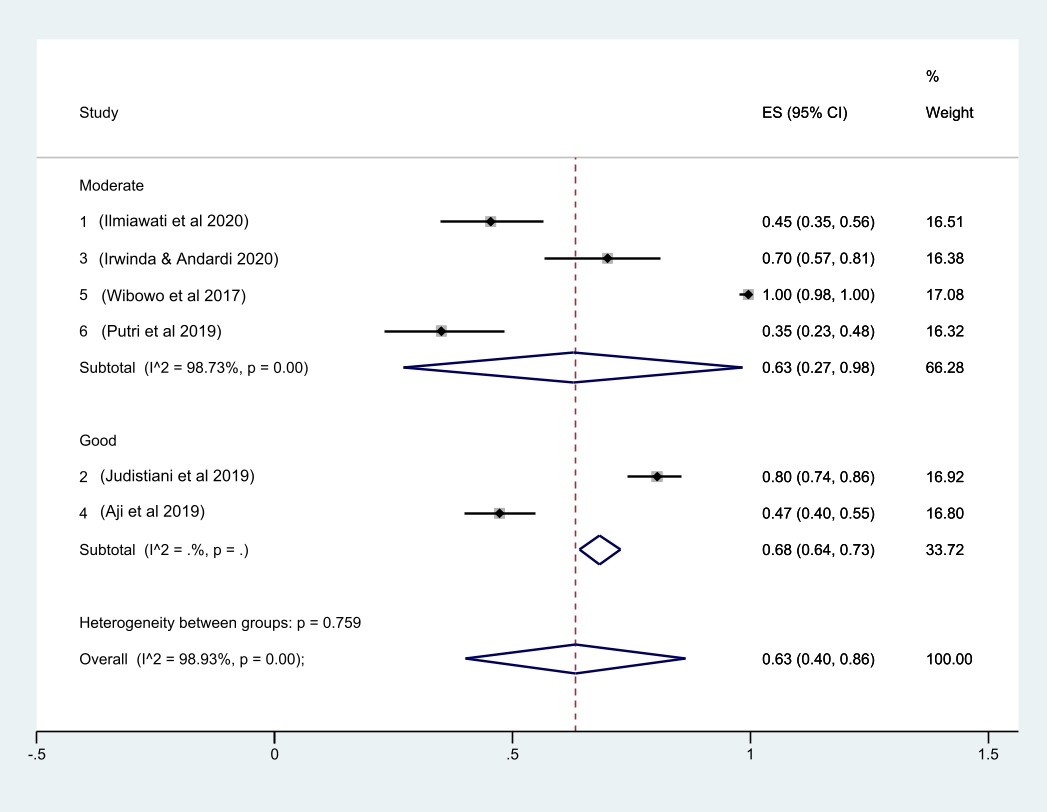


Supplementary Figure 4. Forest plot of vitamin D deficiency according to Newcastle Ottawa Scale criteria


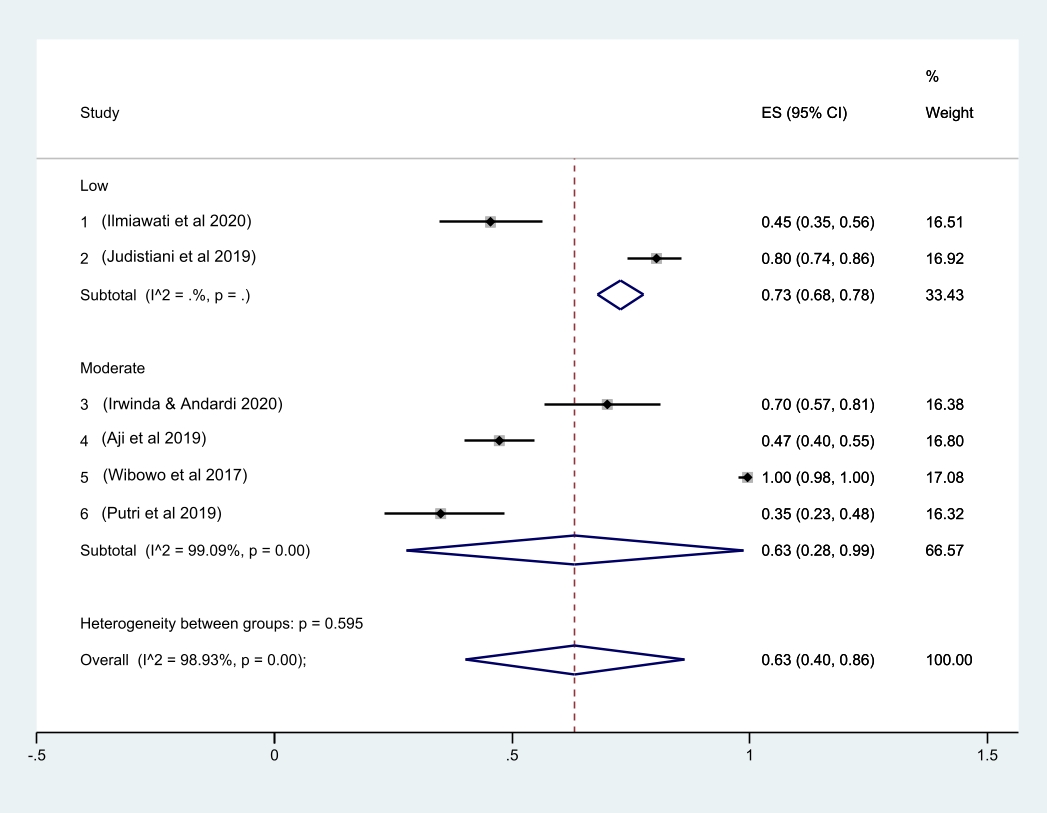


Supplementary Figure 5. Forest plot of vitamin D deficiency according to Joanna Briggs Institute criteria


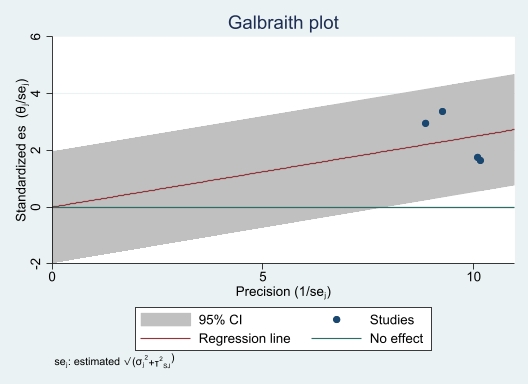


Supplementary Figure 6. Galbraith plot of vitamin D insufficiency


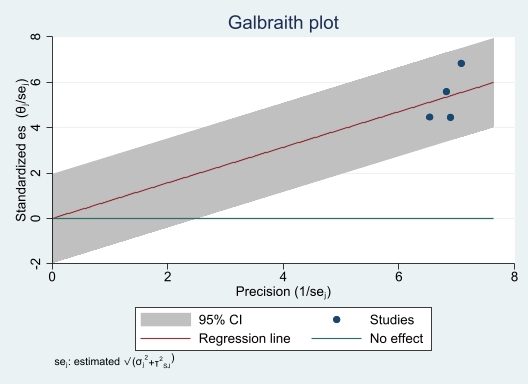


Supplementary Figure 7. Galbraith plot of hypovitaminosis D


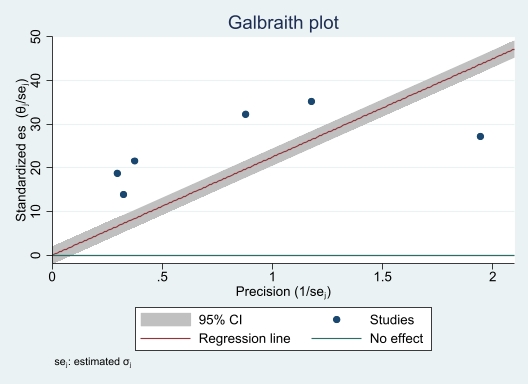


Supplementary Figure 8. Galbraith plot of mean vitamin D level
