# Supplementary material for: A systematic review and meta-analysis of prevalence of vitamin D deficiency among Indonesian pregnant women: a public health emergency
Source: AJOG Glob Rep. 2023 Mar 12;3(2):100189. doi: 10.1016/j.xagr.2023.100189 (PMC10205541; doi:10.1016/j.xagr.2023.100189)
Supplement: Supplementary file 2 [file mmc2.docx]

Supplementary Table 1. Medical subject heading (MeSH) terms and keywords used in each database

| Database | Medical subject heading | The number of studies found |
| --- | --- | --- |
| Pubmed | ("pregnancy"[MeSH Terms] OR "pregnancy"[All Fields] OR "pregnancies"[All Fields] OR "pregnancy s"[All Fields]) AND ("indonesia"[MeSH Terms] OR "indonesia"[All Fields] OR "indonesia s"[All Fields] OR "indonesias"[All Fields]) AND ("vitamin d deficiency"[MeSH Terms] OR "vitamin d deficiency"[All Fields] OR (("vitamin d"[MeSH Terms] OR "vitamin d"[All Fields] OR "ergocalciferols"[MeSH Terms] OR "ergocalciferols"[All Fields]) AND ("insufficiencies"[All Fields] OR "insufficiency"[All Fields] OR "insufficient"[All Fields] OR "insufficiently"[All Fields])) OR ("rickets"[MeSH Terms] OR "rickets"[All Fields] OR "hypovitaminosis d"[All Fields]) OR ("ergocalciferols"[MeSH Terms] OR "ergocalciferols"[All Fields] OR "ergocalciferol"[All Fields]) OR ("25 hydroxyvitamin d"[Supplementary Concept] OR "25 hydroxyvitamin d"[All Fields] OR "25 hydroxyvitamin d"[All Fields] OR "calcifediol"[MeSH Terms] OR "calcifediol"[All Fields])) | 19 |
| Medline | (("vitamin d deficiency"[MeSH Terms] OR "vitamin d deficiency"[All Fields]) AND ("pregnancy"[MeSH Terms] OR "pregnancy"[All Fields])) AND ("indonesia"[MeSH Terms] OR "indonesia"[All Fields]) | 225 |
| Cochrane Library | (Vitamin D):ti,ab,kw AND (Pregnant): Ti,ab,kw | 12 |
| Google Scholar | allintitle: Vitamin D AND Pregnant "Indonesia | 117 |
| Science Direct | (Vitamin D Deficiency OR Vitamin D insufficiency) AND pregnancy AND Indonesia | 352 |
| Indonesian Scientific Journal Database* | Vitamin D dan hamil | 0 |
| Neliti | Vitamin D and pregnancy | 13 |
|  | Vitamin D dan hamil | 3 |
| Indonesia One Search | Vitamin D and pregancy in Indonesia | 16 |
| MedRxiv & BioRxiv | Vitamin D Deficiency OR Vitamin D insufficiency OR hypovitaminosis D AND Pregnancy AND Indonesia | 120 |

*Database does not allow the search to be conducted using Boolean terms

Supplementary Table 2. Assessment of risk of bias using the Joanna Briggs Institute (JBI) Checklist

| Author | JBI 1 | JBI 2 | JBI 3 | JBI 4 | JBI 5 | JBI 6 | JBI 7 | JBI 8 | JBI 9 | Total JBI score | Overall risk of bias |
| --- | --- | --- | --- | --- | --- | --- | --- | --- | --- | --- | --- |
| Ilmiawati et al. (2020)^(1)^ | N | N | Y | Y | Y | Y | Y | Y | U | 3 | Low |
| Judistiani et al (2019)^(2)^ | Y | U | Y | Y | Y | Y | Y | Y | N | 2 | Low |
| Irwinda & Andardi (2020)^(3)^ | Y | N | Y | Y | U | N | Y | Y | U | 4 | Moderate |
| Aji et al. (2019)^(4)^ | U | N | Y | Y | Y | U | Y | Y | N | 4 | Moderate |
| Wibowo et al (2017)^(5)^ | U | U | Y | Y | N | U | Y | Y | N | 5 | Moderate |
| Putri et al (2019)^(6)^ | U | U | U | Y | N | N | Y | Y | N | 6 | Moderate |

Y = Yes; U= Unclear; N = No

Supplementary Table 3. Notable excluded studies with reasoning

| Studies | Reasons for exclusions |
| --- | --- |
| Hutabarat et al. (2018)^(7)^ | Sample size less than 50 |
| Irwinda et al. (2019)^(8)^ |  |
| Judistiani et al. (2018)^(9)^ | Data comes from the same database |
| Judistiani et al. (2019)^(10)^ |  |
| Dhamayanti et al. (2019)^(11)^ |  |
| Wibowo et al. (2016)^(12)^ |  |
| Yuniati et al. (2019)^(13)^ |  |
| Irianti et al. (2020)^(14)^ |  |
| Juwita et al. (2021)^(15)^ |  |
| Wibowo et al. (2015)^(16)^ |  |
| Oktaria et al. (2021)^(17)^ | No data on maternal vitamin D |
| Wahyuningsih et al. (2021)^(18)^ | Not enough data to assess the prevalence |
| Sunarno et al. (2020)^(19)^ |  |
| Aji et al. (2020)^(20)^ | Studies came from the same cohort  (Vitamin D Pregnant Mother [VDPM]) |
| Aji et al. (2020)^(21)^ |  |
| Aji et al. (2019)^(22)^ |  |
| Aji et al. (2018)^(23)^ |  |
| Aji et al. (2019)^(24)^ |  |

Supplementary References

1. Ilmiawati C, Oviana A, Friadi A, Reza M. Sunlight exposed body surface area is associated with serum 25-hydroxyvitamin D (25(OH)D) level in pregnant Minangkabau women, Indonesia. BMC Nutr. 2020;6:18.

2. Judistiani RTD, Nirmala SA, Rahmawati M, Ghrahani R, Natalia YA, Sugianli AK, et al. Optimizing ultraviolet B radiation exposure to prevent vitamin D deficiency among pregnant women in the tropical zone: report from cohort study on vitamin D status and its impact during pregnancy in Indonesia. BMC Pregnancy Childbirth. 2019;19(1):209.

3. Irwinda R, Andardi B. Lower placental 25-hydroxyvitamin D(3) (25(OH)D(3)) and higher placental CYP27B1 and 25(OH)D(3) ratio in preterm birth. J Nutr Sci. 2020;9:e50.

4. Aji AS, Yusrawati Y, Malik SG, Lipoeto NI. The Association of Maternal Vitamin D Status during Pregnancy and Neonatal Anthropometric Measurements: A Longitudinal Study in Minangkabau Pregnant Women, Indonesia. J Nutr Sci Vitaminol. 2020:S63-S70.

5. Wibowo N, Bardosono S, Irwinda R, Syafitri I, Putri AS, Prameswari N. Assessment of the nutrient intake and micronutrient status in the first trimester of pregnant women in Jakarta. Medical Journal of Indonesia. 2017;26:109-15.

6. Putri NI, Lipoeto NI, Rita RS, Aji AS. Hubungan Kadar Vitamin D pada Ibu Hamil dengan Berat Bayi Lahir di Kabupaten Tanah Datar dan Kabupaten Solok. Jurnal Ilmiah Universitas Batanghari Jambi. 2019;19:61-4.

7. Hutabarat M, Wibowo N, Obermayer-Pietsch B, Huppertz B. Impact of vitamin D and vitamin D receptor on the trophoblast survival capacity in preeclampsia. PLoS One. 2018;13(11):e0206725.

8. Irwinda R, Wibowo N, Putri AS. The Concentration of Micronutrients and Heavy Metals in Maternal Serum, Placenta, and Cord Blood: A Cross-Sectional Study in Preterm Birth. J Pregnancy. 2019;2019:5062365.

9. Judistiani RTD, Gumilang L, Nirmala SA, Irianti S, Wirhana D, Permana I, et al. Association of Colecalciferol, Ferritin, and Anemia among Pregnant Women: Result from Cohort Study on Vitamin D Status and Its Impact during Pregnancy and Childhood in Indonesia. Anemia. 2018;2018:2047981.

10. Judistiani RTD, Madjid TH, Irianti S, Natalia YA, Indrati AR, Ghozali M, et al. Association of first trimester maternal vitamin D, ferritin and hemoglobin level with third trimester fetal biometry: result from cohort study on vitamin D status and its impact during pregnancy and childhood in Indonesia. BMC Pregnancy and Childbirth. 2019;19(1):112.

11. Dhamayanti M, Noviandhari A, Supriadi S, Judistiani RTD, Setiabudiawan B. Association of maternal vitamin D deficiency and infants' neurodevelopmental status: A cohort study on vitamin D and its impact during pregnancy and childhood in Indonesia. Journal of Paediatrics and Child Health. 2020;56(1):16-21.

12. Wibowo N, Bardosono S, Irwinda R. Effects of Bifidobacterium animalis lactis HN019 (DR10TM), inulin, and micronutrient fortified milk on faecal DR10TM, immune markers, and maternal micronutrients among Indonesian pregnant women. Asia Pac J Clin Nutr. 2016;25(Suppl 1):S102-s10.

13. Yuniati T, Judistiani RTD, Natalia YA, Irianti S, Madjid TH, Ghozali M, et al. First trimester maternal vitamin D, ferritin, hemoglobin level and their associations with neonatal birthweight: Result from cohort study on vitamin D status and its impact during pregnancy and childhood in Indonesia. J Neonatal Perinatal Med. 2020;13(1):63-9.

14. Irianti S, Judistiani RTD, Rachmayati S, Effendi JS, Setiabudiawan B. Relationship between Maternal Vitamin D Level and Small for Gestational Age Infant in West Java, Indonesia. Majalah Kedokteran Bandung. 2020;52:180-4.

15. Juwita F, Gumilang L, Risan NA, Dhamayanti M. The Association of Vitamin D and Neurodevelopmental Status Among 2 Years Old Infants. Global Pediatric Health. 2021;8:2333794X211034075.

16. Wibowo N, irwinda R. The effect of multi-micronutrient and protein supplementation on iron and micronutrients status in pregnant women. Medical Journal of Indonesia. 2015;24:168-75.

17. Oktaria V, Danchin M, Triasih R, Soenarto Y, Bines JE, Ponsonby A-L, et al. The incidence of acute respiratory infection in Indonesian infants and association with vitamin D deficiency. PLOS ONE. 2021;16(3):e0248722.

18. Wahyuningsih D, Usman AN, Prihantono. Analysis of serum levels L-arginine and 25-hydroxyvitamin D as a predictor of survival of severe preeclampsia mothers. Gac Sanit. 2021;35 Suppl 2:S224-s6.

19. Sunarno I, Usman AN, Idris I, Arsyad MA. Low serum levels of 25-hydroxyvitamin D in severe preeclampsia: The need for early supplementation. Enfermería Clínica. 2020;30:581-4.

20. Aji AS, Erwinda E, Rasyid R, Yusrawati Y, Malik SG, Alathari B, et al. A genetic approach to study the relationship between maternal Vitamin D status and newborn anthropometry measurements: the Vitamin D pregnant mother (VDPM) cohort study. J Diabetes Metab Disord. 2020;19(1):91-103.

21. Aji AS, Yusrawati Y, Malik SG, Lipoeto NI. The Association between Vitamin D-Related Gene Polymorphisms and Serum 25-Hydroxyvitamin D Concentration: A Prospective Cohort Study in Pregnant Minangkabau Women, Indonesia. J Nutr Sci Vitaminol. 2020;66:S295-303.

22. Aji AS, Yerizel E, Desmawati D, Lipoeto NI. Low Maternal Vitamin D and Calcium Food Intake during Pregnancy Associated with Place of Residence: A Cross-Sectional Study in West Sumatran Women, Indonesia. Open Access Macedonian Journal of Medical Sciences. 2019;7(17):2879-85.

23. Aji AS, Yerizel E, Desmawati, Lipoeto NI. The association between lifestyle and maternal vitamin D during pregnancy in West Sumatra, Indonesia. Asia Pac J Clin Nutr. 2018;27(6):1286-93.

24. Aji AS, Erwinda E, Yusrawati Y, Malik SG, Lipoeto NI. Vitamin D deficiency status and its related risk factors during early pregnancy: a cross-sectional study of pregnant Minangkabau women, Indonesia. BMC Pregnancy and Childbirth. 2019;19(1):183.
